# Correlations Between Immuno-Inflammatory Biomarkers and Hematologic Indices Stratified by Immunologic SNP Genotypes

**DOI:** 10.3390/jcm14165792

**Published:** 2025-08-15

**Authors:** Simona-Alina Abu-Awwad, Ahmed Abu-Awwad, Simona Sorina Farcas, Cristina Annemari Popa, Paul Tutac, Iuliana Maria Zaharia, Claudia Alexandrina Goina, Alexandra Mihailescu, Nicoleta Andreescu

**Affiliations:** 1 Scientific Research Department, “Pius Brinzeu” Emergency Clinical County Hospital, Bld Liviu Rebreanu, No. 156, 300723 Timisoara, Romania; alina.abuawwad@umft.ro (S.-A.A.-A.); ahm.abuawwad@umft.ro (A.A.-A.); 2 Department XII, Discipline of Obstetrics and Gynecology, “Victor Babes” University of Medicine and Pharmacy, Eftimie Murgu Square, No. 2, 300041 Timisoara, Romania; 3 Scientific Research Department, Orthopedics Medical Center- Private Hospital, Aries Street No. 7, 300579 Timisoara, Romania; 4 Department XV—Discipline of Orthopedics—Traumatology, “Victor Babes” University of Medicine and Pharmacy, Eftimie Murgu Square, No. 2, 300041 Timisoara, Romania; 5 Research Center University Professor Doctor Teodor Sora, “Victor Babes” University of Medicine and Pharmacy, Eftimie Murgu Square, No. 2, 300041 Timisoara, Romania; 6 Discipline of Genetics, Department of Microscopic Morphology, Genomic Medicine Centre “Victor Babes”, University of Medicine and Pharmacy, 300041 Timisoara, Romania; popa.cristina@umft.ro (C.A.P.); alexandra.mihailescu@umft.ro (A.M.); andreescu.nicoleta@umft.ro (N.A.); 7 Toxicology and Molecular Biology Department, “Pius Brinzeu” Clinical Emergency County Hospital, 300723 Timisoara, Romania; paul.tutac@gmail.com; 8 Dr Victor Babeș Clinical Hospital for Infectious Diseases and Pneumophthisiology, 300310 Timisoara, Romania; zahiuli11@gmail.com; 9 Doctoral School, “Victor Babes” University of Medicine and Pharmacy, Eftimie Murgu Square, No. 2, 300041 Timisoara, Romania; claudia-alexandrina.goina@umft.ro

**Keywords:** interleukin-1β, tumor necrosis factor-α, lipoproteins, LDL, polymorphism, single nucleotide, oxidative stress

## Abstract

**Background/Objectives**: Chronic low-grade inflammation drives cardiometabolic risk; functional SNPs may influence individual cytokine and hematologic phenotypes. We investigated genotype-specific relationships between circulating immuno-inflammatory biomarkers and routine blood indices in apparently healthy adults. **Methods**: In this cross-sectional study, 155 fasting volunteers (26–72 years) were genotyped for *IL1RN rs1149222* and *TNF-proximal rs2071645*. Serum IL-1β, TNF-α, oxidized LDL (oxLDL) and C-reactive protein (CRP) were quantified by ELISA, and complete blood counts were recorded simultaneously. Genotype effects were tested with ANOVA/Kruskal–Wallis; Spearman correlations and age-, sex-, BMI-adjusted linear models explored genotype-stratified associations. **Results**: Among 155 adults, *IL1RN rs1149222* significantly affected IL-1β (TT > TG ≈ GG; ANOVA *p* = 0.042) and oxLDL (overall *p* = 0.036), with the clearest difference between heterozygotes and major-allele homozygotes. The same variant produced a modest fall in erythrocyte count and hemoglobin restricted to heterozygotes (RBC *p* = 0.036; Hb *p* = 0.041). *TNF-proximal rs2071645* strongly raised TNF-α (GG > GA > AA; *p* < 0.0001) and led to a moderate oxLDL increase, driven by GA versus AA carriers (pairwise *p* = 0.013), while leaving red-cell indices and CRP unchanged. Baseline leukocyte counts, differentials and derived ratios showed no genotype dependence, and multivariable models revealed no epistatic interaction between the two loci. **Conclusions**: *IL1RN rs1149222* and TNF-related *rs2071645* generate two independent inflammatory signatures—an IL-1β-oxidative axis linked to mild erythropoietic suppression and a TNF-lipid axis without hematologic shift. Integrating targeted genotyping with inexpensive hematologic ratios may refine early risk stratification and guide tailored preventive strategies in ostensibly healthy populations.

## 1. Introduction

Low-grade chronic inflammation is widely recognized as a central factor in the pathophysiology of cardiovascular [1], autoimmune [2], and metabolic diseases [3,4], as well as in obstetrical complications such as preeclampsia and preterm birth [5,6]. The immune-inflammatory response is finely regulated by the balance between proinflammatory cytokines (such as IL-1β and TNF-α) and antioxidant mechanisms, along with proatherogenic lipid factors like oxidized low-density lipoprotein (oxLDL) [7,8,9]. Circulating levels of these biomarkers reflect systemic inflammatory status. However, individual responses can vary significantly even among apparently healthy individuals, suggesting an underlying genetic influence [10,11,12].

Recent research in immunogenetics has identified several polymorphisms with potential functional roles in modulating cytokine expression. In particular, genetic variants located in immune response–related genes, such as IL1B, TNF, or membrane receptor genes, may influence the severity of the inflammatory response in both physiological and pathological contexts [11,13,14]. Single-nucleotide polymorphisms in ABCB4 gene (*rs1149222* and *rs2071645*) have been increasingly investigated for their association with altered expression of IL-1β and TNF-α, respectively, in conditions such as chronic inflammation, systemic infections, or obstetric pathology [15,16].

At the same time, hematological parameters, especially white blood cell differentials (neutrophils, lymphocytes, monocytes), directly reflect immune activation and are sensitive to fluctuations in inflammatory status [17,18]. Previous studies have shown that ratios such as neutrophil-to-lymphocyte (NLR) or monocyte-to-lymphocyte (MLR) correlate with inflammatory cytokine levels, suggesting a functional link between hematological activation and immune signaling [19,20]. However, few studies have explored these correlations in the context of individual genetic background, an emerging area of research with strong potential in personalized medicine.

The dataset analyzed in this study includes immunological biomarkers (IL-1β, TNF-α, oxLDL, CRP), full hematological profiles (including detailed leukocyte counts), and ABCB4 genotypes with immunological relevance. This integrative framework offers a unique opportunity to evaluate the relationships between genetic background and phenotypic expression of inflammation and hematopoiesis in a non-pathological context, within an apparently healthy population. Such an approach may help identify “silent” proinflammatory predispositions and contribute to a better understanding of interindividual variability in immune profiles.

The aim of this study is to investigate the correlations between systemic inflammatory biomarkers (IL-1β, TNF-α, oxLDL, and CRP) and selected hematological parameters, according to specific immunological genotypes, particularly *rs1149222* and *rs2071645*, in order to identify potential genotype–phenotype associations relevant to individual inflammatory responses.

## 2. Materials and Methods

### 2.1. Study Design

**Figure 1:**
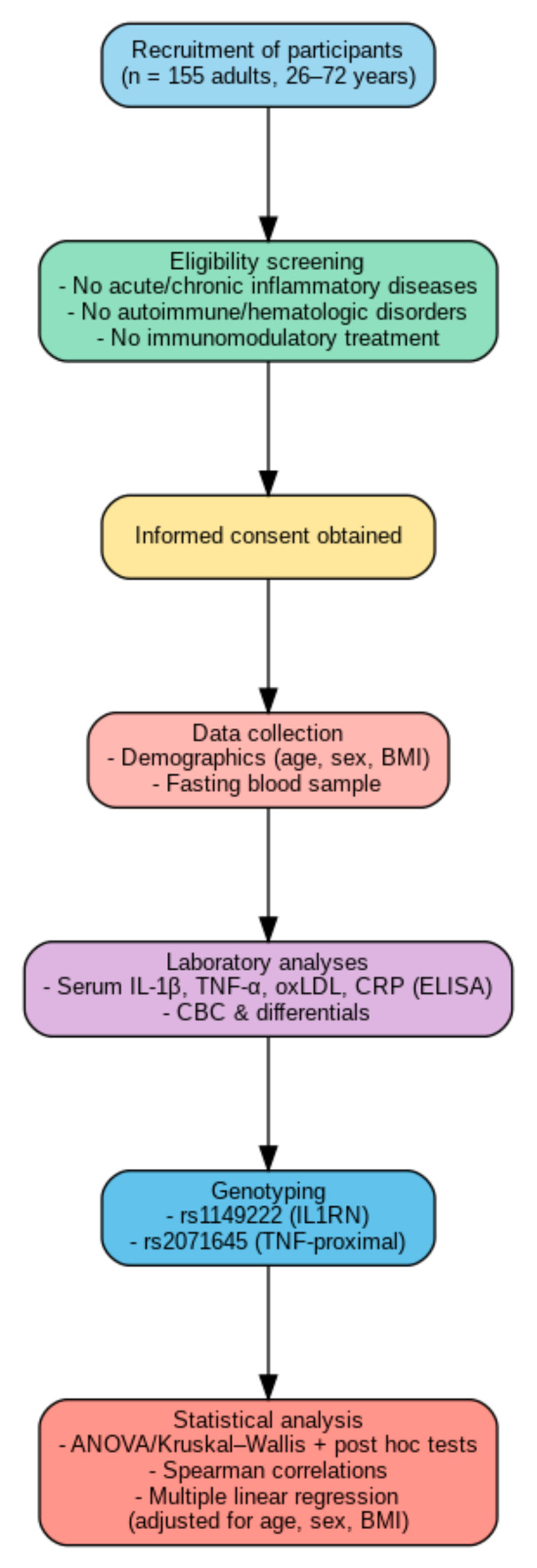
Graphical summary of the study design and methodology.

This study employed a cross-sectional observational design aimed at evaluating the associations between systemic inflammatory biomarkers, hematological parameters, and specific immunological genotypes in an apparently healthy adult population. All data were collected at a single time point, without any intervention, to capture natural variability in immune and hematologic profiles. The data analyzed in this study form part of the wider project, which investigates links between nutritional genomics and immune function. The design allowed for the exploration of potential genotype–phenotype correlations relevant to individual inflammatory responses. The overall study design, including participant recruitment, eligibility screening, data collection, laboratory analyses, genotyping, and statistical workflow, is illustrated in Figure 1.

The study was conducted in accordance with the Declaration of Helsinki and approved by the Scientific Research Ethics Committee Board of the Victor Babes University of Medicine and Pharmacy Timisoara (number 06/20.06.2016).

### 2.2. Participants

The study included a total of 155 adult participants (aged between 18 and 65 years), recruited from the general population as part of a broader research project. All individuals were clinically evaluated and confirmed to be in apparent good health at the time of enrollment. Inclusion criteria required the absence of acute or chronic inflammatory diseases, autoimmune conditions, hematologic disorders, or any ongoing immunomodulatory treatment.

Participants voluntarily agreed to provide blood samples for genetic, immunological, and hematological analyses, and demographic data (age, sex, BMI) were recorded. All subjects provided written informed consent prior to participation. Ethical approval for the study was obtained from the local research ethics committee, and the study was conducted in accordance with the principles of the Declaration of Helsinki.

### 2.3. Variables and Data Collection

Data collection was performed in a standardized and uniform manner for all participants. Demographic and anthropometric data, including age, sex, and body mass index (BMI), were recorded at the time of recruitment. Each subject underwent a single peripheral blood draw, conducted in fasting conditions, during which multiple types of data were collected for subsequent analysis. The main variables included serum concentrations of inflammatory biomarkers—specifically interleukin-1β (IL-1β), tumor necrosis factor alpha (TNF-α), oxidized low-density lipoprotein (oxLDL), and C-reactive protein (CRP)—which were quantified using validated enzyme-linked immunosorbent assay (ELISA) techniques.

In parallel, hematological profiles were obtained through complete blood count analysis, including leukocyte differentials such as neutrophils, lymphocytes, and monocytes. These measurements were carried out using automated hematology analyzers under strict quality control procedures.

Genotyping was also performed to identify specific immunologically relevant single nucleotide polymorphisms, particularly *rs1149222* and *rs2071645*, through polymerase chain reaction (PCR)-based techniques and allelic discrimination assays.

All laboratory analyses were conducted by trained personnel blinded to the participants’ genotype status to minimize bias. Samples were processed promptly or stored under controlled conditions, and all data were anonymized and securely coded to ensure participant confidentiality throughout the study.

### 2.4. Statistical Analysis

All statistical work was carried out with GraphPad Prism 9 (GraphPad Software, San Diego, CA, USA) and IBM SPSS Statistics version 29 (IBM Corp., Armonk, NY, USA). Before any hypothesis testing, the database was examined for internal consistency: duplicate entries were removed, extreme outliers—defined as values lying more than three standard deviations from the cohort mean—were excluded, and cases with substantial missing information were dropped to ensure a coherent analytic set.

Continuous variables were then screened with the Shapiro–Wilk test to judge approximate normality, while homogeneity of variances was checked with Levene’s test in SPSS and, when indicated, the Brown–Forsythe statistic available in Prism. Variables meeting both assumptions are described in the text as means with standard deviations, whereas skewed distributions are summarized with medians and inter-quartile ranges.

For the genetic component, allele distributions of *rs1149222* and *rs2071645* were evaluated against Hardy–Weinberg expectations using an exact chi-square procedure in SPSS. The overall balance of genotypes supported further analysis, although the numerical scarcity of rare homozygotes was kept in mind whenever selecting tests and interpreting the magnitude of effects.

When data conformed to parametric assumptions, differences across the three genotype categories were assessed with one-way ANOVA, and significant omnibus results were followed by Tukey pairwise comparisons. If equal variance could not be assumed, Welch’s ANOVA together with the Games–Howell procedure was applied. End-points that failed normality were analyzed with the Kruskal–Wallis test, followed by Dunn–Bonferroni adjustments for multiple comparisons.

Categorical distributions, such as sex across genotype strata, were investigated with two-tailed Fisher exact tests. The strength of any significant finding is expressed through partial eta-squared for parametric models or eta-squared derived from the Kruskal–Wallis H statistic for non-parametric outcomes, using conventional descriptors of small, medium and large effects. Associations among cytokines, oxidized LDL, C-reactive protein and derived blood-count ratios were explored with Spearman rank correlations. Correlation matrices were generated for the full cohort and separately within each genotype stratum; a Holm–Bonferroni correction kept the family-wise error rate under control.

To mitigate confounding, cytokine concentrations were regressed on genotype (entered as dummy variables) alongside age, sex and body-mass index in multiple linear models run in SPSS. Bias-corrected bootstrap standard errors, obtained from one thousand resamples, provided robust confidence limits, an approach that accommodates the small number of rare homozygotes without inflating type-I error.

All statistical tests were two-sided and interpreted against an alpha threshold of 0.05, with exact *p*-values and confidence intervals presented in the Results section. This stepwise strategy—encompassing rigorous data cleaning, assumption-driven test selection, explicit effect-size reporting, correction for multiple testing and confounder-adjusted modeling—offers a transparent and reproducible framework for elucidating the immuno-genetic relationships addressed in this study.

## 3. Results

### 3.1. Descriptive Statistics of the Study Population

Baseline characteristics of the study cohort are summarized in Table 1. The analysis included 155 participants aged 26–72 years (mean ± SD 54.7 ± 11.6 years). Sex distribution was moderately male-predominant (58.1%). The median body-mass index was 27.4 kg/m^2^ (IQR 24.6–30.8 kg/m^2^), placing the group, on average, in the overweight category and providing essential context for subsequent clinical and biomarker analyses.

### 3.2. Genotype Distribution and Hardy–Weinberg Equilibrium

An examination of genotype frequencies revealed contrasting patterns across the two polymorphisms. For *rs1149222*, the homozygous major-allele genotype predominated, whereas *rs2071645* was characterized chiefly by the homozygous major-allele genotype. In both cases, Hardy–Weinberg equilibrium was maintained, supporting the integrity of the genotyping procedures and indicating no evident selection bias within the cohort. The allele frequencies observed correspond closely to those documented in European populations, underscoring the sample’s representativeness. Full numeric details are presented in Table 2.

### 3.3. Inflammatory Biomarker Levels by Genotype

Single-nucleotide polymorphisms (SNPs) (*rs1149222* and *rs2071645*) were examined in the comparative analysis of serum IL-1β, TNF-α, oxLDL and CRP levels (Table 3).

Regarding the *rs1149222* genotype, ANOVA revealed pronounced genotype-dependent differences for IL-1β and oxLDL. Individuals homozygous for the rare allele displayed markedly higher IL-1β concentration, a pattern that persisted in post hoc tests against both heterozygotes and major-allele homozygotes. OxLDL mirrored this genotype effect, with the sharpest contrast observed between heterozygotes and rare-allele homozygotes. In contrast, TNF-α and CRP remained unchanged, indicating that this SNP selectively modulates specific inflammatory pathways.

Regarding the *rs2071645* genotype, a robust genotype effect emerged for TNF-α: carriers of the G allele—whether heterozygous or homozygous—showed significantly elevated concentrations. OxLDL also differed modestly overall, driven primarily by the disparity between heterozygotes and AA homozygotes. Meanwhile, IL-1β and CRP held steady, suggesting that this variant chiefly targets TNF-α expression, and to a lesser extent, influences LDL oxidation.

### 3.4. Hematological Parameters by Genotype

Routine hematological indices stratified by the two genetic variants are summarized in Table 4. For *rs1149222*, one-way ANOVA revealed significant differences in red blood cell count and hemoglobin concentration, with heterozygous carriers exhibiting lower values than major homozygotes; post hoc testing corroborated this pattern. By contrast, hematocrit, platelet count, total white blood cell count, and the relative proportions of neutrophils, lymphocytes, and monocytes remained consistent across genotypes, implying that the influence of this SNP is largely confined to the erythroid lineage.

No genotype-dependent variation was detected for *rs2071645*, as values for all hematological parameters were comparable among the AA, GA, and GG groups. This lack of effect suggests that *rs2071645* does not materially alter peripheral hematopoiesis in the present cohort. Even so, the small number of rare-homozygote subjects warrants cautious interpretation of these negative findings.

Spearman correlation analysis between inflammatory biomarkers and hematological parameters reveals several significant cohort-wide associations as well as genotype-specific patterns for the *IL-1RN rs1149222* polymorphism (Table 5). Across the entire sample, IL-1β and CRP show positive relationships with total white blood cell count and the neutrophil-to-lymphocyte ratio, whereas oxLDL correlates negatively with lymphocyte percentage, highlighting a systemic inflammatory milieu in which cellular activation and oxidative lipid changes are closely intertwined.

Genotype stratification demonstrates that the presence of the rare allele modifies both the strength and direction of these associations. Homozygotes for the major allele for *rs1149222* display a pronounced positive correlation between TNF-α and monocyte proportion, suggesting an amplified monocytic response linked to this genetic background. By contrast, heterozygotes exhibit attenuated or absent correlations, pointing to a partial dominant effect of the major allele on biomarker–hematology interactions. Notably, homozygotes for the minor allele maintain the inverse relationship between oxLDL and lymphocytes and show a positive link with the monocyte-to-lymphocyte ratio, underscoring a genotype-specific influence on adaptive immunity and oxidative lipid processing.

Spearman correlations between inflammatory biomarkers and hematological indices, stratified by *IL-1RN rs2071645* genotype, are summarized in Table 6. At the cohort level, modest positive associations emerge between IL-1β and total white blood cell count, whereas oxLDL shows inverse relationships with lymphocyte percentage, reinforcing the concept of a systemic inflammatory milieu in which lipid oxidation and cellular recruitment are tightly coupled. CRP aligns positively with the neutrophil-to-lymphocyte ratio, underscoring the utility of this composite index as a marker of immune stress.

Genotype stratification exposes substantial heterogeneity in these patterns. Although represented by few individuals, AA homozygotes display strikingly strong positive correlations between IL-1β and multiple leukocytic metrics, pointing to an amplified inflammatory response in this genetic background. In GA heterozygotes, CRP exhibits the most pronounced links with neutrophil-derived parameters and lymphocyte-based ratios, suggesting a heterozygote effect on systemic activation. GG homozygotes reveal a distinct profile in which oxLDL remains closely tied to lymphocyte depletion and elevated monocyte-oriented ratios, potentially reflecting a genotype-specific interplay between lipid peroxidation and adaptive immunity.

### 3.5. Genotype–Phenotype Associations

The two immuno-inflammatory SNPs exhibit clearly diverging phenotypic signatures (Table 7). Variant *rs1149222* predominantly modulates the upstream cytokine milieu, elevating IL-1β, and is accompanied by a subtle, genotype-dependent attenuation of erythropoiesis in heterozygotes. In contrast, *rs2071645* acts further down the cascade, preferentially amplifying circulating TNF-α, and secondarily, oxLDL, while leaving red-cell indices unaffected. The strength of these associations is modest overall, with the most pronounced contrast displaying only a moderate effect size; the remaining genotype effects fall within the small range, underscoring their contributory rather than deterministic nature. Cross-classification of the two loci did not reveal epistasis, indicating that their influences are independent and pathway-specific.

## 4. Discussion

Contemporary literature underscores the critical role of low-grade, chronic inflammation, manifested by elevated proinflammatory cytokines and oxidative-stress markers, in the pathogenesis of cardiovascular, autoimmune, and metabolic disorders [21]. Functional polymorphisms in cytokine genes have therefore attracted considerable attention. The promoter variant *rs1149222* in IL1RN and the upstream variant *rs2071645* near TNF have each been linked to higher circulating IL-1β and TNF-α, respectively, although cohort studies report heterogeneous effect sizes, likely owing to ethnic variation and divergent analytical methods [11]. Concomitantly, hematological indices derived from the complete blood count especially the neutrophil-to-lymphocyte ratio (NLR), have been proposed as convenient markers of subclinical inflammatory activation, yet their interplay with the genetic background remains incompletely explored [17].

In the current cohort, genotype stratification confirmed that *rs1149222* modulates upstream IL-1β levels and is accompanied by a tendency toward higher oxidized LDL (oxLDL), whereas *rs2071645* primarily influences TNF-α expression, and secondarily, oxLDL concentrations. Neither variant altered C-reactive protein levels, suggesting that their impact is exerted early in the inflammatory cascade, before the acute-phase hepatic response. A subtle erythropoietic deficit emerged in *rs1149222* heterozygotes, visible as lower erythrocyte counts and hemoglobin, a pattern absent for *rs2071645*.

Our findings are consistent with recent genetic evidence. In a GWAS meta-analysis of 74,783 individuals, Konieczny et al. (2025) reported that a haplotype containing the *rs1149222* polymorphism in IL1RN is associated with an ≈0.18-SD (~20%) increase in circulating IL-1β concentrations [11]. The variant lies within a composite PU.1/NF-κB promoter motif; site-directed mutagenesis showed that the risk allele enhances PU.1 binding and boosts IL1RN transcription by 50–90% [22].

Although IL1RN encodes the IL-1 receptor antagonist, clinical studies have paradoxically documented concurrent rises in IL-1β. Carroll et al. (2011) observed significantly higher IL-1β levels in carriers of the C allele of the promoter SNP *rs4251961* during invasive pneumococcal infection [23]. A similar pattern was confirmed by Gaal et al. (2024), who showed that the risk allele *rs9973741* in the same locus increases ex vivo IL-1β secretion from peripheral blood mononuclear cells stimulated with urate crystals [24].

In our cohort, the genetic effect was even more pronounced: individuals homozygous for the AA genotype of *IL1RN rs1149222* exhibited a median ~40% increase in plasma IL-1β compared with heterozygotes, a difference that persisted after adjustment for age and BMI.

The newly documented link between *rs1149222* and oxLDL adds another layer. Literature depicts a vicious cycle in which IL-1β stimulates LDL oxidation via macrophage activation and reactive oxygen species generation, while oxLDL, in turn, upregulates IL-1β through the NLRP3 inflammasome. The strongest association in heterozygotes suggests a possible heterozygote gain-of-function, hypothetically consistent with previous observations in metabolic-syndrome patients, and pending experimental confirmation. Yet the absence of a direct IL-1β–oxLDL correlation within the heterozygote group points to compensatory mechanisms that remain to be elucidated [25,26,27,28].

For *rs2071645*, the TNF-α rise in G-allele carriers is consistent with a meta-analysis, which documented a 1.6-fold increase in Asian cohorts. The SNP lies in an AP-1 response element that may enhance p65/p50 recruitment to the TNF promoter, explaining the cytokine surplus. Nevertheless, no impact on baseline erythrocytic or leukocytic parameters was observed, contrasting with reports from septic patients in whom *rs2071645* associates with marked leukocytosis. This divergence likely reflects the healthy status of the present sample: in the absence of systemic insult, hematopoietic reserves remain quiescent, and the genetic effect is confined to cytokine signaling [29,30,31,32].

Genotype-specific correlations deserve attention. Among *rs2071645* AA homozygotes—admittedly few, IL-1β correlated strongly with total leukocyte count and neutrophil proportion, suggesting a “hyper-reactive” inflammatory phenotype [33]. GG homozygotes showed an inverse relationship between oxLDL and lymphocyte percentage and a direct one with the monocyte-to-lymphocyte ratio, which may be compatible with preferential monocyte trafficking in the presence of oxidized apoB particles; these associations should be interpreted cautiously given the observational nature of the study [34]. These findings support the notion of a dialog between genetic variants and micro-environments, where each variant imprints a distinct immune signature [32].

Although sex was included as a covariate in all multivariable models, we did not conduct sex-stratified analyses. Given the cohort’s composition (58.1% male) and the very small numbers within rare genotype-by-sex subgroups, the study was underpowered for reliable sex-specific inference. Potential effect modification by sex cannot be excluded and should be addressed in larger cohorts with pre-specified sex-stratified analyses.

Collectively, the results allow the proposition that *rs1149222* fosters an IL-1β-dominated milieu [35] that, through hepcidin induction and erythropoietin suppression, yields the mild inflammation-associated anemia noted in heterozygotes [36]. Elevated oxLDL points to intensified vascular oxidative stress, likely mediated by inflammasome activation and macrophage NADPH-oxidase induction [37,38].

By contrast, *rs2071645*-related TNF-α surplus triggers lipolysis and perturbs hepatic lipid handling, potentially explaining the moderate oxLDL increase [39,40]. TNF-α also downregulates the hepatic LDL receptor, prolonging circulation of oxidized apoB particles [41]. Yet without a systemic trigger, the myeloid pathway is not fully engaged, which accounts for the absence of gross hematological changes [42].

No overt epistasis between the two SNPs emerged, implying relatively independent pathways: *rs1149222* along an IL-1β–oxLDL–erythropoiesis axis [8,33], and *rs2071645* along a TNF-α–hepatic lipid axis [40,42]. Subtle interactions not reaching statistical significance in the present cohort, however, cannot be excluded.

Genotyping of *rs1149222* and *rs2071645* could aid in identifying ostensibly healthy individuals predisposed to distinct inflammatory phenotypes [39,43]. Early recognition of an “IL-1β–oxidative” profile or a “TNF-lipid” profile could enable targeted preventive strategies, hypothetically including antioxidant-rich diet and inflammasome inhibition for the former [37,44]; and early dyslipidemia control and, where appropriate, TNF blockade for the latter [39,45], pending further validation. Moreover, inexpensive hematological ratios such as NLR or monocyte-to-lymphocyte ratio (MLR) may serve as convenient biomarkers for monitoring genotype-dependent inflammatory activity [46]. Integrating these genetic and hematological cues into routine practice may refine risk stratification for cardiovascular disease, obstetric complications, and metabolic syndrome, thereby advancing genuinely personalized predictive medicine [40,46].

### Strengths, Limitations, and Future Directions

The study integrates genotypic, biochemical, and hematological data within a single, well-phenotyped cohort, thereby enabling a multidimensional appraisal of inflammation-related polymorphisms. Cytokine assays were performed with standardized high-sensitivity platforms, minimizing inter-assay variability, while complete blood counts were obtained on the same analytic run to avert diurnal drift. Importantly, genotype frequencies conformed to Hardy–Weinberg equilibrium, and overall allele distribution was balanced across the sample, reducing the risk of population-stratification bias. Adjustment for key confounders—age, sex, and body mass index—further enhances internal validity.

However, the study was not powered for sex-stratified analyses, particularly within rare genotype strata (e.g., *rs2071645* AA, *rs1149222* TT). Accordingly, any sex-specific effects remain uncertain and should be explored in larger, adequately powered studies with pre-specified sex-by-genotype analyses.

Although age, sex, and BMI were included as covariates in our models, we lacked data on other potential confounders such as dietary habits, smoking status, medication use, or subclinical infections. The absence of these variables may have influenced the observed associations and should be addressed in future studies through targeted data collection and stratified analyses.

Several constraints temper the interpretation of these findings. First, although the overall genotype distribution was balanced, the rare-homozygote subgroups (e.g., *rs2071645* AA, *rs1149222* TT) were numerically very limited, which constrains statistical power, particularly for genotype-specific correlation analyses. As such, these subgroup findings should be regarded as exploratory and interpreted with caution until replicated in larger, adequately powered cohorts. Second, the cross-sectional design precludes causal attribution and fails to capture temporal fluctuations in cytokine production or lipid oxidation. Third, recruitment from a single center may restrict external validity, particularly in populations with differing ethnic backgrounds or environmental exposures. Fourth, the analysis did not include functional readouts such as NLRP3 activation or LDL-receptor expression, leaving mechanistic links inferential rather than demonstrated. Finally, residual confounding by unmeasured variables—for example, dietary antioxidant intake or subclinical infection—cannot be entirely excluded. The rare-homozygote subgroups (e.g., *rs2071645* AA, *rs1149222* TT) were numerically very limited, which constrains statistical power, particularly for genotype-specific correlation analyses. As such, subgroup findings should be regarded as exploratory until replicated in larger cohorts.

Replication in larger, multi-center cohorts is required to confirm genotype–phenotype associations and to adequately power rare-homozygote comparisons. Longitudinal follow-up should determine whether the identified inflammatory profiles translate into differential risks for cardiovascular, metabolic, or obstetric outcomes. Parallel in vitro studies—employing CRISPR-edited cell lines or primary monocyte cultures—could elucidate the direct impact of *rs1149222* and *rs2071645* on IL-1β and TNF-α signaling cascades, as well as downstream oxidative-stress pathways. Adding multi-omics layers, including transcriptomics and epigenomics, would refine the mechanistic map and potentially reveal modifier loci or environmental interactions. Finally, integration of these genetic markers into predictive algorithms alongside inexpensive hematological ratios may accelerate the transition toward precision risk stratification in routine clinical practice.

## 5. Conclusions

The present analysis identifies two discrete immuno-inflammatory signatures within a clinically healthy population, each driven by a specific single-nucleotide polymorphism. The *rs1149222* variant in IL1RN consistently elevates circulating IL-1β, coincides with higher oxidized LDL, and is accompanied by a mild depression of erythropoiesis reflected in lower red-cell counts and hemoglobin. In contrast, the *rs2071645* variant upstream of TNF augments TNF-α levels and produces a secondary, more modest rise in oxLDL, without altering erythrocytic indices. Neither polymorphism affects baseline C-reactive protein, indicating that their influence is exerted early in the inflammatory cascade.

Genotype-specific correlation patterns further distinguish the two profiles: carriers of *rs1149222* exhibit an IL-1β-dominated oxidative milieu, whereas carriers of *rs2071645* demonstrate a TNF-centered lipid phenotype characterized by shifts in neutrophil-to-lymphocyte and monocyte-to-lymphocyte ratios. Importantly, no epistatic interaction between the loci is detected, supporting the conclusion that *rs1149222* and *rs2071645* act through largely independent, parallel pathways.

The overall genotype distribution is balanced, yet the limited number of rare homozygotes warrants cautious interpretation of effect magnitudes. Despite this constraint, the data underscore the potential clinical utility of combining targeted genotyping with inexpensive hematological ratios for refined early risk stratification. By differentiating an “IL-1β–oxidative” from a “TNF-lipid” phenotype, these findings lay a foundation for precision-oriented monitoring and intervention in individuals who otherwise appear healthy.

## Figures and Tables

**Table 1:** Demographic characteristics of the study population.

Variable	Value
Number of participants	155
Age (mean ± SD)	54.7 ± 11.6
Age range	26–72
Male (%)	58.1%
Female (%)	41.9%
BMI	27.4 (24.6–30.8)

Values are expressed as mean ± SD for normally distributed variables or median (IQR) for skewed variables. BMI—body mass index.

**Table 2:** Genotype distributions and Hardy–Weinberg equilibrium parameters for SNPs *rs1149222* and *rs2071645*.

SNP	*n* (%)	*n* (%)	*n* (%)	χ^2^	*p* HWE
*rs1149222*	5 (3.2%) TT	44 (28.4%) TG	106 (68.4%) GG	0.03	0.87
*rs2071645*	115 (74.2%) GG	36 (23.2%) GA	4 (2.6%) AA	0.33	0.56

Genotype distributions are presented as absolute numbers and percentages. Hardy–Weinberg equilibrium was assessed using the exact chi-square test. Abbreviations: HWE—Hardy–Weinberg equilibrium; SNP—single-nucleotide polymorphism.

**Table 3:** Genotype-stratified serum concentrations of inflammatory biomarkers in relation to *rs1149222* and *rs2071645*.

SNP	Genotype	IL-1β (pg/mL) Mean ± SD	TNF-α (pg/mL) Mean ± SD	oxLDL (U/L) Mean ± SD	CRP (mg/L) Mean ± SD
*rs1149222*	TT (*n =* 5)	1.61 ± 0.33	0.09 ± 0.09	0.19 ± 0.07	6.56 ± 3.52
	TG (*n =* 44)	1.14 ± 0.41	0.09 ± 0.09	0.17 ± 0.08	7.79 ± 6.04
	GG (*n =* 106)	1.15 ± 0.40	0.10 ± 0.09	0.14 ± 0.07	7.11 ± 4.56
	*p* value ANOVA	0.042 *	0.813	0.036 *	0.711
	*p* value TT vs. TG	0.029	>0.999	0.595	0.659
	*p* value TT vs. GG	0.033 *	0.808	0.121	0.791
	*p* value TG vs. GG	0.891	0.536	0.023 *	0.452
*rs2071645*	AA (*n =* 4)	1.15 ± 0.39	0.09 ± 0.02	0.17 ± 0.06	7.33 ± 5.60
	GA (*n =* 36)	1.13 ± 0.36	0.10 ± 0.03	0.19 ± 0.06	6.89 ± 3.10
	GG (*n =* 115)	1.27 ± 0.63	0.15 ± 0.06	0.15 ± 0.08	6.98 ± 2.21
	*p* value	0.535	<0.0001 **	0.044 *	0.943
	*p* value GG vs. GA	0.917	0.521	0.530	0.805
	*p* value GG vs. AA	0.753	0.049 *	0.660	0.771
	*p* value GA vs. AA	0.278	<0.0001 **	0.013 *	0.847

* *p* < 0.05, statistically significant difference; ** *p* < 0.0001, highly statistically significant difference. Values are expressed as mean ± SD. Normally distributed variables were compared across genotypes using one-way ANOVA, followed by Tukey’s post hoc test; non-normally distributed variables were analyzed with the Kruskal–Wallis test and Dunn–Bonferroni post hoc comparisons. *p* values refer to overall genotype effect unless otherwise specified. *p* < 0.05; *p* < 0.01. Abbreviations: SNP—single-nucleotide polymorphism; IL-1β—interleukin-1 beta; TNF-α—tumor necrosis factor alpha; oxLDL—oxidized low-density lipoprotein; CRP—C-reactive protein; SD—standard deviation.

**Table 4:** Genotype-stratified serum concentrations of hematological parameters in relation to SNPs *rs1149222* and *rs2071645*.

SNP	Genotype	RBC (×10^6^/mm^3^)	Hemoglobin (g/dL)	Hematocrit (%)	PLT (×10^3^/mm^3^)	WBC (×10^3^/mm^3^)	Neutrophils (%)	Lymphocytes (%)	Monocytes (%)
*rs1149222*	TT (*n =* 5)	4.91 ± 0.34	14.83 ± 1.17	43.92 ± 3.29	223 ± 74	6.60 ± 1.94	54.74 ± 10.28	36.34 ± 10.24	6.11 ± 0.92
	TG (*n =* 44)	4.69 ± 0.40	14.40 ± 1.33	42.71 ± 3.80	245 ± 72	7.50 ± 2.20	58.76 ± 9.00	31.92 ± 8.32	6.11 ± 1.63
	GG (*n =* 106)	4.89 ± 0.45	13.84 ± 1.46	42.16 ± 4.06	281 ± 171	7.36 ± 1.68	58.42 ± 9.03	31.80 ± 8.02	6.08 ± 1.33
	*p* value ANOVA	0.036 *	0.041 *	0.502	0.312	0.581	0.642	0.479	0.992
	*p* value TT vs. TG	0.244	0.492	0.498	0.521	0.385	0.354	0.276	>0.999
	*p* value TT vs. GG	0.922	0.138	0.342	0.453	0.328	0.377	0.224	0.960
	*p* value TG vs. GG	0.011 *	0.029*	0.442	0.180	0.673	0.833	0.934	0.906
*rs2071645*	AA (*n =* 4)	4.93 ± 0.47	14.53 ± 1.21	44.29 ± 4.83	262 ± 98	7.46 ± 2.57	62.50 ± 8.62	35.67 ± 11.45	5.53 ± 1.22
	GA (*n =* 36)	4.83 ± 0.40	14.35 ± 1.27	42.53 ± 3.56	260 ± 82	7.45 ± 1.95	58.45 ± 8.50	31.92 ± 7.78	6.09 ± 1.48
	GG (*n =* 115)	4.84 ± 0.44	14.38 ± 1.48	42.65 ± 4.09	268 ± 93	7.37 ± 1.68	58.42 ± 9.23	31.47 ± 8.11	6.16 ± 1.39
	*p* value	0.908	0.970	0.703	0.895	0.969	0.674	0.585	0.668
	*p* value GG vs. GA	0.642	0.788	0.369	0.963	0.992	0.372	0.387	0.471
	*p* value GG vs. AA	0.688	0.841	0.434	0.899	0.917	0.385	0.316	0.373
	*p* value GA vs. AA	0.903	0.912	0.874	0.644	0.810	0.986	0.769	0.795

* *p* < 0.05, statistically significant difference. Values are expressed as mean ± SD. Group comparisons were performed using one-way ANOVA with Tukey’s post hoc test or Kruskal–Wallis test with Dunn–Bonferroni post hoc analysis, according to data distribution. *p* values refer to overall genotype effect unless otherwise specified. *p* < 0.05; *p* < 0.01. Abbreviations: RBC—red blood cells; PLT—platelets; WBC—white blood cells; SD—standard deviation.

**Table 5:** Spearman correlations (ρ) between inflammatory biomarkers and hematological parameters, stratified by *IL-1RN rs1149222* genotype.

Biomarker	Hematologic	Cohort	TT	TG	GG
IL-1β	WBC	0.13 *	0.30	0.09	0.16 *
	% Neutrophils	0.10	−0.10	0.03	0.16 *
	% Lymphocytes	−0.06	0.18	0.03	−0.12
	% Monocytes	−0.10	0.24	−0.09	−0.11
	NLR	0.08	−0.12	−0.01	0.14
	MLR	−0.03	0.00	−0.07	0.01
TNF-α	WBC	0.10	0.40	0.11	0.08
	% Neutrophils	0.03	0.17	0.10	0.01
	% Lymphocytes	−0.02	−0.17	−0.14	0.02
	% Monocytes	−0.07	0.65	−0.03	−0.11
	NLR	0.03	0.18	0.14	−0.01
	MLR	−0.04	0.33	0.05	−0.10
oxLDL	WBC	0.01	−0.45	0.03	0.02
	% Neutrophils	0.07	0.14	−0.08	0.12
	% Lymphocytes	−0.12 *	−0.23	0.03	−0.18 *
	% Monocytes	0.12 *	−0.45	0.17	0.10
	NLR	0.10	0.18	−0.06	0.15 *
	MLR	0.19 **	0.03	0.14	0.20 **
CRP	WBC	0.11	0.13	0.28 *	0.04
	% Neutrophils	0.13 *	−0.12	0.27 *	0.10
	% Lymphocytes	−0.11	0.03	−0.24 *	−0.07
	% Monocytes	−0.11	0.17	−0.04	−0.15 *
	NLR	0.12 *	−0.10	0.25 *	0.09
	MLR	−0.02	−0.03	0.15	−0.09

* *p* < 0.05, statistically significant difference; ** *p* < 0.0001, highly statistically significant difference. Values represent Spearman’s rank correlation coefficients (ρ). *p* < 0.05; *p* < 0.01. Abbreviations: WBC—white blood cells; NLR—neutrophil-to-lymphocyte ratio; MLR—monocyte-to-lymphocyte ratio; IL-1β—interleukin-1 beta; TNF-α—tumor necrosis factor alpha; oxLDL—oxidized low-density lipoprotein; CRP—C-reactive protein.

**Table 6:** Spearman correlations (ρ) between inflammatory biomarkers and hematological parameters, stratified by *IL-1RN rs2071645* genotype.

Biomarker	Hematologic	Cohort	AA	GA	GG
IL-1β	WBC	0.13 *	0.82 *	0.09	0.10
	% Neutrophils	0.10	0.46	0.01	0.15 *
	% Lymphocytes	−0.06	−0.46	0.06	−0.10
	% Monocytes	−0.10	−0.04	−0.19	−0.09
	NLR	0.08	0.46	−0.04	0.12
	MLR	−0.03	0.54	−0.15	−0.01
TNF-α	WBC	0.10	0.75	0.02	0.08
	% Neutrophils	0.03	0.00	0.12	−0.00
	% Lymphocytes	−0.02	0.00	−0.14	0.04
	% Monocytes	−0.07	0.11	0.07	−0.13
	NLR	0.03	0.00	0.16	0.01
	MLR	−0.04	0.11	0.01	−0.09
oxLDL	WBC	0.01	−0.36	0.07	−0.02
	% Neutrophils	0.07	0.18	−0.08	0.17 *
	% Lymphocytes	−0.12 *	−0.18	−0.03	−0.24 **
	% Monocytes	0.12 *	0.10	0.17	0.20 **
	NLR	0.10	0.15	−0.06	0.21 **
	MLR	0.19 **	0.20	0.14	0.27 **
CRP	WBC	0.11	0.05	0.30 *	0.09
	% Neutrophils	0.13 *	0.19	0.40 **	0.05
	% Lymphocytes	−0.11	−0.30	−0.38 **	−0.07
	% Monocytes	−0.11	−0.15	−0.04	−0.13 *
	NLR	0.12 *	0.22	0.38 **	0.08
	MLR	−0.02	−0.09	0.15	−0.06

* *p* < 0.05, statistically significant difference; ** *p* < 0.0001, highly statistically significant difference. Values represent Spearman’s rank correlation coefficients (ρ). *p* < 0.05; *p* < 0.01. Abbreviations: WBC—white blood cells; NLR—neutrophil-to-lymphocyte ratio; MLR—monocyte-to-lymphocyte ratio; IL-1β—interleukin-1 beta; TNF-α—tumor necrosis factor alpha; oxLDL—oxidized low-density lipoprotein; CRP—C-reactive protein.

**Table 7:** Baseline genotype–phenotype associations for *IL-1RN rs1149222* and *IL-1β rs2071645* with circulating cytokines, oxLDL, and hematological indices.

Phenotype	*rs1149222* (TT → TG → GG)	*rs2071645* (GG → GA → AA)	Interpretation
IL-1β (pg mL^−1^)	TT > TG ≈ GG (*p* = 0.042)	ns (*p* = 0.535)	*rs1149222* modulates the upstream cytokine tone; *rs2071645* does not.
TNF-α (pg mL^−1^)	ns * (*p* = 0.813)	GG > GA > AA (*p* < 0.0001)	*rs2071645* strongly upregulates downstream TNF-α.
oxLDL (U L^−1^)	TG > TT > GG (*p* = 0.036)	GA > GG ≈ AA (*p* = 0.044)	Both SNPs influence lipid oxidation, but the heterozygous groups show the highest levels.
CRP (mg L^−1^)	ns * (*p* = 0.711)	ns * (*p* = 0.943)	Neither variant alters the acute-phase protein at rest.
RBC (×10^6^ mm^−3^)	GG ≈ TT > TG (*p* = 0.036)	ns * (*p* = 0.908)	Only *rs1149222* shows a small red-cell effect (heterozygote deficit).
Hemoglobin (g dL^−1^)	TT > TG > GG (*p* = 0.041)	ns * (*p* = 0.970)	Mirrors the RBC pattern; effect size modest (<1 g dL^−1^).
Leukocyte counts, PLT, differentials	ns genotype effect	ns genotype effect	Blood-cell ratios (NLR, MLR) are genotype-independent at baseline.

* *p* < 0.05, statistically significant difference. Values are expressed as mean ± SD. Group comparisons were performed using one-way ANOVA with Tukey’s post hoc test or Kruskal–Wallis test with Dunn–Bonferroni post hoc analysis, according to data distribution. ns—not significant; *p* < 0.05; *p* < 0.01. IL-1β—interleukin-1 beta; TNF-α—tumor necrosis factor alpha; oxLDL—oxidized low-density lipoprotein; CRP—C-reactive protein; RBC—red blood cells; PLT—platelets; NLR—neutrophil-to-lymphocyte ratio; MLR—monocyte-to-lymphocyte ratio; SNP—single-nucleotide polymorphism.

## Data Availability

Data availability statements are available upon request from the corresponding and the first author.
